# Association Between Neonatal Arrhythmia and Mortality and Recurrence: A Retrospective Study

**DOI:** 10.3389/fped.2022.818164

**Published:** 2022-03-11

**Authors:** Lihong Ran, Jie Li, Lei Bao, Long Chen

**Affiliations:** ^1^Department of Neonatology, Children's Hospital of Chongqing Medical University, Chongqing, China; ^2^Chongqing Key Laboratory of Pediatrics, Chongqing, China; ^3^Ministry of Education Key Laboratory of Child Development and Disorders, Chongqing, China; ^4^National Clinical Research Center for Child Health and Disorders, Chongqing, China; ^5^China International Science and Technology Cooperation Base of Child Development and Critical Disorders, Chongqing, China; ^6^Department of Obstetrics and Gynecology, The First Affiliated Hospital of Chongqing Medical University, Chongqing, China

**Keywords:** neonates, arrhythmia, neonatal intensive care unit, mortality, recurrence, follow-up

## Abstract

**Objective:**

The aim of the present study was to explore the association between neonatal arrhythmia (NA) and mortality and recurrence.

**Methods:**

A single-center, retrospective study was performed between January 1, 2015, and December 31, 2019. Neonates with NA were eligible and divided into either benign or non-benign groups. The primary outcomes were mortality and recurrence within 2 to 6 years.

**Results:**

NA was diagnosed in 189 patients (0.39%) after screening 47,911 hospitalized neonates, and 10 of them did not finish the follow-up. Finally, 179 neonates were included into the final analysis (58 in the non-benign NA and 121 in the benign NA groups). The incidences of death and recurrence for NA were 5.59% (10/179) and 18.44% (33/179). Compared with neonates with benign NA, those neonates with non-benign NA were shown higher rates of death (13.79% vs. 1.65%; odds ratio [OR], 5.73; 95% confidence interval [CI], 1.07–31.01; *p* = 0.04) and recurrence (44.83% vs. 5.79%; OR, 8.49; 95% CI, 3.12–23.08; *p* < 0.001).

**Conclusions:**

Neonates with non-benign NA were shown higher rates of death and recurrence when compared with benign NA. Because of high recurrence, more attention was needed in neonates with non-benign NA after discharge.

## Introduction

Neonatal arrhythmia (NA) is a rare but often severe disorder that typically injures the cardiac conduction path (1–4). The incidence of NA has been estimated between 1% and 10% in all newborns (4–6). The clinical presentations of NA are variable, including asymptomatic and symptomatic with or without congestive heart failure and cardiogenic shock. Most patients present good recovery without sequelae. Some subtypes of NA need long-term antiarrhythmic therapy, especially tachyarrhythmias. Approximately 10% of patients may die due to congestive heart failure (7, 8).

In the past, several studies have been enforced to assess the association between arrhythmia and mortality and recurrence in newborn infants, and the results were inconsistent. Binnetoglu et al. (9) performed a retrospective study in 66 newborns with arrhythmia in a tertiary care hospital, and the results showed that the two most common NAs were supraventricular ectopic beats (39.39%) and supraventricular tachycardia (SVT) (22.72%). Five patients (33.33%) with SVT had a recurrence after the neonatal period, and it persisted beyond the age of 1 year in two patients, and four patients died in the newborn period for other reasons, such as respiratory problems, metabolic disease, and sepsis. Isik et al. (10) also retrospectively analyzed 17 newborns with arrhythmia; the results reported that benign arrhythmias disappeared in the first month of life, and all of the patients with SVT required antiarrhythmic maintenance therapy without recurrence during hospitalization, and one of the patients with SVT died due to chronic lung disease in the second month of life. In the two studies above, no patients died of arrhythmia. In contrast, a retrospective observational study demonstrated that non-benign NA could cause hemodynamic imbalance and increase the risk of death, and the mortality rate was 23.64%, with the ages ranging from 1 to 90 days in the neonatal intensive care unit (NICU) (11).

To date, rare studies reported the association between NA and mortality and recurrence. Therefore, the aim of the present study was to assess the association between NA during the neonatal period and mortality and recurrence in the long-term follow-up.

## Materials and Methods

### Study Design

Neonatal clinical data were retrospectively collected in the tertiary NICU of Children's Hospital of Chongqing Medical University, China, from January 1, 2015, to December 31, 2019. The study was approved by the ethics committee of Children's Hospital of Chongqing Medical University (file no. 2019-195) and registered at ClinicalTrials.Gov (NCT04899596) (registration on May 21, 2021; registration name: High Risk Factor, Clinical Feature, and Follow up of Neonatal Arrhythmia). The trial was performed in accordance with the approved guidelines.

### Inclusion and Exclusion Criteria

#### Inclusion Criteria

Included neonates simultaneously met the following two conditions: (1) occurrence of NA within 28 days of life; (2) abnormal 12-lead electrocardiography (ECG) and/or abnormal 24-h Holter ECG.

#### Exclusion Criteria

If neonates met one of the following two conditions, they should be excluded: (1) major non-cardiac congenital anomalies, including esophageal atresia, annular pancreas, congenital megacolon, congenital anorectal malformations, pulmonary sequestration, congenital bronchogenic cyst of the lung, congenital pulmonary hypoplasia, congenital hydrocephalus, congenital tonsillar hernia deformity, and palatoschisis; (2) chromosomal abnormalities.

### Primary and Secondary Outcomes

The primary outcomes were death and recurrence from birth to August 15–31, 2021. Recurrence was defined as arrhythmia recurrence after discharge. The secondary outcomes were antiarrhythmic therapy, including adenosine triphosphate (0.20–0.40 mg/kg per time, intravenous injection), propafenone (1.00–1.50 mg/kg per time, intravenous injection), metoprolol (1.00–2.00 mg/kg per day, two times a day, oral administration), sotalol (4.00–6.00 mg/kg per day, two times a day, oral administration), propranolol (2.00–4.00 mg/kg per day, two times a day, oral administration), cedilanid (30.00 μg/kg, intravenous injection), Digoxin (10.00 μg/kg per day, per 12 h, oral administration), benazepril (0.10–0.30 mg/kg per day, once a day, oral administration), lidocaine (1.00 mg/kg per time, intravenous injection), and verapamil (0.10–0.20 mg/kg per time, intravenous injection). Combined use of antiarrhythmic drug was defined as a patient receiving antiarrhythmic treatment with equal to or more than two drugs.

### Termination of the Study

The study would end if one of the following conditions was reached: (1) death within August 15–31, 2021; (2) parents' decision not to continue participation.

### Data Collection and Follow-Up

The medical records for all enrolled newborn infants were collected into a database according to the protocol. We also investigated the usefulness of antiarrhythmic therapy, death, and recurrence by telephone on August 15–31, 2021.

### Key Definition

NAs were classified as either benign or non-benign. Benign arrhythmias included sinus arrhythmia, premature atrial contraction (PAC), premature ventricular contraction (PVC), first-degree atrioventricular (AV) block, and junctional arrhythmia. Nonbenign arrhythmias included SVT, ventricular tachycardia (VT), atrial flutter (AFL), ventricular fibrillation, second- or third-degree (or complete) AV block, and genetic arrhythmias such as congenital long-QT syndrome (2, 11).

SVT was the most common type of non-benign NA, defined as tachycardia resulting from an abnormal mechanism involving heart structures proximal to the bifurcation of the bundle of His; usually, the frequency was unvarying, and more than 230 beats/min with an abnormal P-wave axis on the surface ECG. Other types of NA were detailed in the references (1–4, 12, 13).

Other definitions were as follows: congenital heart disease (CHD) included atrial septal defect, patent ductus arteriosus, ventricular septal defect, complete transposition of the great artery, and coarctation of the aorta. Oligoamnios and hydramnios were defined as 5 cm or less and 18 cm or more of amniotic fluid index (14). The diagnoses of sepsis, cardiac damage, heart failure, hemodynamic instability, and shock were based on consensus and guidelines (15–17).

### Sample Size Estimation

The sample size estimation was calculated using PASS v8.0.3 (NCCS, USA). According to previous studies; the mortality in both groups was ~9.7 and 0.3%, respectively. A plausible estimate of the coincidence rate in both groups was 2%. With 80% power and a 2-sided significance level of 0.05, 56 infants would be needed at least in each group.

### Statistical Analysis

Quantitative variables were first checked for normality using Kolmogorov–Smirnov test; results are presented as mean ± standard deviation and compared using two-tailed Student *t* test if the variable was normally distributed and homoscedastic, or median (interquartile range) and compared with Mann-Whitney *U* test if the variable were not normally distributed or not homoscedastic. Qualitative variables were present as numbers and compared using the χ^2^ test or the Fisher test.

The epidemiological data of the enrolled neonates differing between the two groups with a *p* < 0.20 at the univariate analysis would be included in a stepwise multivariate logistic regression model to identify independent risk factors associated with death and recurrence. The probability of stepwise was 0.05 for entry and 0.10 for removal. Model goodness-of-fit would be evaluated with Hosmer–Lemeshow test (*p* > 0.05). We used SPSS v16.0 (SPSS Inc., USA) for statistical analysis. For all analyses, *p* < 0.05 was regarded as significant.

## Results

**Figure 1:**
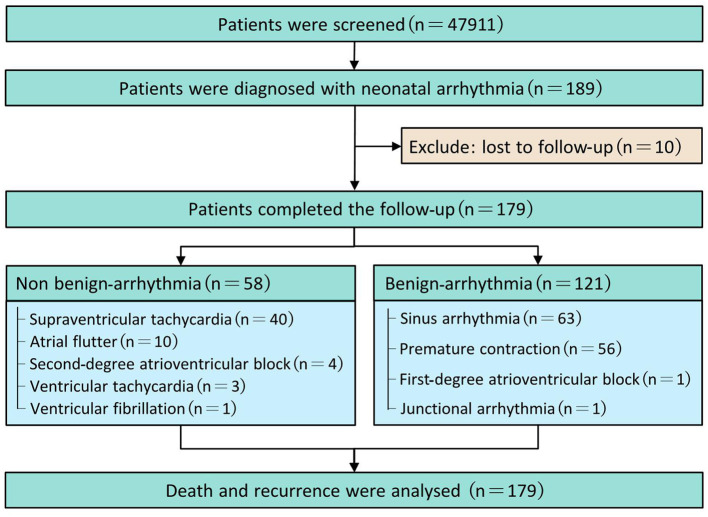
Flow diagram.

From January 1, 2015, to December 31, 2019, NA was diagnosed in 189 patients (0.39%) after screening 47,911 hospitalized neonates. In the telephone follow-up, the median duration of follow-up was 39 months (33–50 months), with 27 months of minimum and 80 months of maximum. Ten neonates were lost to follow-up. Finally, 179 neonates finished the trial (58 in the non-benign arrhythmia group and 121 in the benign arrhythmia group) and were included in the final analysis (Figure 1). Among the 179 patients, 10 (5.59%) died, and 33 (18.44%) had a recurrence, respectively.

Three main non-benign arrhythmia and benign arrhythmia were 68.97% of SVT (40/58), 17.24% of AFL (10/58), and 6.9% of second-degree AV block (4/58) and 52.07% of sinus arrhythmia (63/121), 46.28% of premature contraction (56/121), and 0.83% of first-degree AV block or junctional arrhythmia (1/121), respectively. The baseline characteristics of neonates with NA are presented in Table 1.

**Table 1:** The baseline characteristics of neonates with arrhythmia.

**Variables**	**Total (*n* = 179)**	**NBA (*n* = 58)**	**BA (*n* = 121)**	**OR/MD (95% CI)**	***p* Value**
**Variables of neonates**					
GA, weeks	39.29 (37.71–40.00)	38.14 (35.86–39.71)	39.43 (38.43–40.14)	—	<0.001
Birth weight, kg	3.20 (2.86–3.50)	3.12 (2.50–3.50)	3.23 (2.92–3.50)	—	0.18
Preterm (yes), %	34 (19.00)	20 (34.48)	14 (11.57)	4.02 (1.85–8.75)	<0.001
Male (yes), %	107 (59.78)	35 (60.34)	72 (59.50)	1.04 (0.55–1.96)	0.92
Asphyxia (yes), %	16 (8.94)	8 (13.79)	8 (6.61)	2.26 (0.80–6.36)	0.12
Fetal arrhythmias (yes), %	8 (4.47)	4 (6.90)	4 (3.31)	2.17 (0.52–8.99)	0.48
Electrolyte disturbance (yes), %	48 (26.82)	19 (32.76)	29 (23.97)	1.55 (0.78–3.08)	0.21
Hypoglycemia (yes), %	12 (6.70)	5 (8.62)	7 (5.79)	1.54 (0.47–5.07)	0.70
EOS (yes), %	22 (12.29)	11 (18.97)	11 (9.09)	2.34 (0.95–5.77)	0.06
Cardiac damage (yes), %	97 (54.19)	32 (55.17)	65 (53.72)	1.06 (0.57–1.99)	0.86
Heart failure (yes), %	8 (4.47)	7 (12.07)	1 (0.83)	16.47 (1.98–137.32)	0.003
Hemodynamic instability (yes), %	23 (12.85)	21 (36.21)	2 (1.65)	33.77 (7.56–150.83)	<0.001
Shock (yes), %	6 (3.35)	5 (8.62)	1 (0.83)	11.32 (1.29–99.27)	0.02
CHD (yes), %	137 (76.53)	49 (84.48)	88 (72.73)	2.04 (0.90–4.62)	0.08
Abnormal CM (yes), %	8 (4.47)	3 (5.17)	5 (4.13)	1.31 (0.30–5.70)	1.00
Mechanic ventilation (yes), %	8 (4.47)	8 (13.79)	0 (0.00)	1.16 (1.05–1.29)	<0.001
**Variables of mother**					
GHT (yes), %	5 (2.79)	2 (3.45)	3 (2.48)	1.41 (0.23–8.65)	1.00
GDM (yes), %	23 (12.85)	9 (15.52)	14 (11.57)	1.40 (0.57–3.46)	0.46
Oligoamnios (yes), %	14 (7.82)	5 (8.62)	9 (7.44)	1.17 (0.38–3.68)	1.00
Hydramnios (yes), %	6 (3.35)	1 (1.72)	5 (4.13)	0.41 (0.05–3.57)	0.69
Maternal age, years	27.99 ± 4.79	28.16 ± 4.31	27.91 ± 5.01	0.77 (−1.27–1.76)	0.75
Cesarean (yes), %	107 (59.78)	44 (75.86)	63 (52.07)	2.89 (1.44–5.82)	0.002
**Variables of outcomes**					
Death (yes), %	10 (5.59)	8 (13.79)	2 (1.65)	9.52 (1.95–46.42)	0.003
Recurrence (yes), %	33 (18.44)	26 (44.83)	7 (5.79)	13.23 (5.26–33.27)	<0.001

*NBA, non-benign arrhythmia; BA, benign arrhythmia; GA, gestational age; GHT, gestational hypertension; GDM, gestational diabetes mellitus; EOS, early-onset sepsis; CHD, congenital heart disease; CM, cardiac markers; OR, odds ratio; MD, mean difference; CI, confidence interval*.

### Primary Outcomes

In the univariate analysis, compared with the benign arrhythmia group, the non-benign arrhythmia group had lower gestational age (GA) (*p* < 0.05), more cesarean section (*p* < 0.05), more mechanic ventilation (*p* < 0.05), more death (*p* < 0.05), more recurrence (*p* < 0.05), and higher incidence of heart failure (*p* < 0.05), shock (*p* < 0.05), and hemodynamic instability (*p* < 0.05).

Compared with the benign arrhythmia group, the non-benign arrhythmia group had a higher incidence of hyperkalemia (25.86% vs. 12.4%; 95% confidence interval [CI], 1.11–5.48; *p* = 0.02). No difference in electrolyte disturbance was shown between the two groups (*p* > 0.05).

There were 145 term and 34 preterm infants (1 of <28 weeks' GA, 28 weeks' GA ≤ 1 <32 weeks' GA, 32 weeks' GA ≤ 4 <34 weeks' GA, 28 of ≥34 weeks' GA). Compared with the benign arrhythmia group, the non-benign arrhythmia group showed a higher incidence of preterm (*p* < 0.05).

In the multivariate logistic regression model, non-benign arrhythmia (*p* < 0.05) and cardiac failure (*p* < 0.05) were the independent risk factors for mortality, and non-benign arrhythmia (*p* < 0.05) and hemodynamic instability (*p* < 0.05) were independent risk factors for recurrence (Table 2).

**Table 2:** Independent risk factors using binary logistic analysis.

**Primary outcomes**	**Risk factors**	**Univariate analysis**				**Multivariate analysis**			
		**CF**	**B**	**OR (95% CI)**	***p***	**CF**	**B**	**OR (95% CI)**	***p***
Death	NBA	10.21	2.25	9.52 (1.95–46.42)	0.005	4.71	1.75	5.76 (1.07–31.01)	0.04
	Heart failure	14.05	3.31	27.50 (5.51–137.24)	<0.001	14.05	2.61	13.53 (2.47–74.21)	0.003
Recurrence	NBA	37.82	2.58	13.23 (5.26–33.27)	<0.001	37.38	2.14	8.49 (3.12–23.08)	<0.001
	Hemodynamic instability	24.72	2.42	11.22 (4.27–29.44)	<0.001	5.81	1.31	3.70 (1.26–10.86)	0.02

*NBA, non-benign arrhythmia; CF, Cohen f^2^; B, regression coefficient β; OR, odds ratio; CI, confidence interval*.

### Antiarrhythmic Therapy and Follow-Up

Forty-four patients of non-benign NA and four of benign NA received antiarrhythmic therapy and are summarized in Table 3. Antiarrhythmic therapy was performed in 44 (75.86%) of the non-benign NA group. The other 14 did not receive antiarrhythmic therapy, including four patients with second-degree AV block, three patients with AFL (all of whom presented a short duration of attack and stopped spontaneously without recurrence), seven patients with SVT (four patients presented a short duration of attack and stopped after stimulation, three patients whose parents gave up treatment because of other diseases and died). Four patients of benign NA received antiarrhythmic therapy, including one patient with junctional arrhythmia (who was treated with propafenone and propranolol and had a recurrence once after discharge, and the patient took propafenone once and metoprolol for 6 months), two patients with PAC (one patient was treated with metoprolol; another one was treated with cedilanid), and one patient with PVC (who was treated with metoprolol). The other 117 in the benign NA group did not receive antiarrhythmic therapy and also presented good recovery.

**Table 3:** Antiarrhythmic therapy and follow-up.

**No**	**Types**	**Antiarrhythmic therapy**												**Antiarrhythmic time after discharge**	**Recurrence (time)**	**Death time**	**Causes of death**
		**ATP**	**Propafenone**	**Metoprolol**	**Sotalol**	**Propranolol**	**Cedilanid**	**Digoxin**	**Benazepril**	**Lidocaine**	**Verapamil**	**Cardioversion**	**Defibrillation**				
1^*^	JR		√	√		√								6 mo	1	—	—
2^*^	PAC						√							0	0	—	—
3^*^	PAC			√										10 d	0	—	—
4^*^	PVC			√										10 d	0	—	—
5	SVT			√				√						10 d	0	—	—
6	SVT			√				√						10 d	0	—	—
7	SVT	√	√	√			√							36 mo	5	—	—
8	SVT		√	√			√	√			√			18 mo	0	—	—
9	SVT			√				√						6 mo	2	—	—
10	SVT		√					√						0	1	—	—
11	SVT	√					√	√						12 mo	10	—	—
12	SVT			√			√	√				√		10 d	0	—	—
13	SVT	√												0	1	—	—
14	SVT			√										1 mo	0	—	—
15	SVT							√						18 mo	2	—	—
16	SVT		√	√				√						0	1	—	—
17	SVT	√	√				√				√			24 mo	5	—	—
18	SVT	√	√					√						18 mo	1	—	—
19	SVT	√												8 mo	3	—	—
20	SVT	√	√											10 d	0	—	—
21	SVT	√												22 mo	4	—	—
22	SVT	√	√	√			√							17 mo	4	—	—
23	SVT	√												0	0	—	—
24	SVT	√		√										10 d	0	—	—
25	SVT			√										10 d	0	—	—
26	SVT			√										6 mo	0	—	—
27	SVT	√												0	0	—	—
28	SVT			√				√			√			24 mo	2	—	—
29	SVT	√		√										0	0	—	—
30	SVT		√		√	√		√						18 mo	15	—	—
31	SVT	√		√										0	1	—	—
32	SVT			√										1 mo	0	—	—
33	SVT	√		√			√							3 mo	1	—	—
34	SVT			√				√						0	0	1.67 mo	Septic shock
35	SVT			√				√				√		10 d	1	2.50 mo	Arrhythmia
36	SVT						√							0	0	15 d	TGA
37	SVT			√				√	√					2 mo	1	2 mo	Coarctation of the aorta
38	AFL			√										1 mo	20	6 mo	Respiratory failure
39	AFL		√	√				√						2 mo	1	—	—
40	AFL	√					√	√						2 mo	0	—	—
41	AFL		√									√		0	0	—	—
42	AFL		√	√										0	1	—	—
43	AFL			√										1 mo	0	—	—
44	AFL			√										12 mo	0	—	—
45	VF									√			√	10 d	0	—	—
46	VT		√											10 d	0	—	—
47	VT												√	0	0	—	—
48	VT		√						√					1 mo	1	—	—

*JR, junctional arrhythmia; PAC, premature atrial contraction; PVC, premature ventricular contraction; SVT, Supraventricular tachycardia; AFL, atrial flutter; VT, ventricular tachycardia; VF, ventricular fibrillation; AV, atrioventricular; ATP, adenosine triphosphate; TGA, complete transposition of the great artery*.

* *Benign NA group*.

Ten patients died (5.59% [10/179]), including seven patients with SVT (three of complex CHD, two of septic shock, two of arrhythmia), one patient with AFL due to respiratory failure, and two patients with sinus arrhythmia (one of respiratory failure, one of complex CHD).

## Discussion

In this single-center, retrospective study, the aim of the present study was to explore the association between arrhythmia during the neonatal period and mortality and recurrence within 39 months of median follow-up. As a result, we found, compared with neonates with benign arrhythmia, those neonates with non-benign arrhythmia showed higher rates of mortality (13.79% vs. 1.65%, odds ratio [OR]: 5.73; 95% CI, 1.07–31.01; *p* = 0.04) and recurrence (44.83% vs. 5.79%; OR, 8.49; 95% CI, 3.12–23.08; *p* < 0.001). Our results indicated that more attention was needed in neonates with non-benign NA after discharge due to the high recurrence rate.

To date, several studies have investigated the relationships between NA and mortality and recurrence, and the results were inconsistent. The study by Binnetoglu et al. (9) reported the recurrence of NA was 7.58% (5/66), and four patients died due to sepsis, respiratory problem, and metabolic disease. Kundak et al. (11) also performed a retrospective observational study, and the result showed that the mortality rate within 3 months was 23.63% (13/55). In the study by Gilljam et al. (8), 109 patients were retrospectively reviewed, and the result showed the mortality was 6.42% (7/109), and the rate of recurrence was 11.93% (13/109), where seven patients died due to heart failure, premature, structural heart disease, and pulmonary vascular disease. Moura et al. (5) performed a retrospective analysis of clinical files in 26 neonates with arrhythmia, and the result showed that one patient died due to cerebral hemorrhage, and two patients presented an episode of SVT after treatment withdrawal. The studies referring to associations between NA and death and recurrence are summarized in Table 4. In the present study, 10 patients died (5.59%), including 8 patients of the non-benign NA group (2 of septic shock, 2 of SVT, 3 of complicated CHD, 1 of respiratory diseases) and two patients of the benign NA group (1 of respiratory disease, 1 of complicated CHD), and the incidence of recurrence was 18.44% (33/179).

**Table 4:** Summary of other NA study referring to the main results.

**References**	**Follow-up (mo)** **Median (min–max)**	**NBA (n)**			**BA (n)**		
		**Total**	**Death**	**Recurrence**	**Total**	**Death**	**Recurrence**
Binnetoglu et al. (9)	15.00 (3.00–72.00)	31	2 of SVT died because of respiratory problem, 1 of complete AV block died because of sepsis	5 of SVT	35	1 of PAC died because of metabolic disease	—
Kundak et al. (11)	—	55	3 of SVT, 4 of complete AV block, 4 of VT, 2 of mixed-type arrhythmia	—	—	—	—
Isik et al. (10)	—	9	1 of SVT died because of chronic lung disease	3 of SVT	8	0	0
Moura et al. (5)	30.80 (0.00–71.00)	26	1 of complete AV block died because of cerebral hemorrhage	2 of SVT	—	—	—
Gilljam et al. (8)	6.00 (0.00–189.60)	109	7 of SVT (4 of heart failure, 1 of premature, 2 of structural heart disease, 1 of pulmonary vascular disease)	13 of SVT	—	—	—
Mou et al. (18)	—	46	3	—	271	0	—
Wang et al. (19)	3.00	38	1 of SVT died because of heart failure, 2 of VT died because of shock	—	51	0	—
Casey et al. (20)	Median 23.00	18	2 of AFL	0	—	—	—
Davis et al. (21)	25.20 (0.00–169.20)	40	6 of VT	—	—	—	—
Eliasson et al. (22)	38.40 (1.00–97.20)	175	14 of complete AV block	—	—	—	—
Total	10 literatures	547	53	23	365	1	0

*NA, neonatal arrhythmia; NBA, non-benign arrhythmia; BA, benign arrhythmia; SVT, supraventricular tachycardia; VT, ventricular tachycardia; AFL, atrial flutter; AV, atrioventricular; PAC, premature atrial contraction*.

NA was mainly presented in term infants. Badrawi et al. (6) performed a prospective observational study in 457 neonates, and the result showed that there was no NA that occurred in preterm infants with GA <32 weeks, whereas 75% of NAs occurred in infants with GA at more than 37 weeks. The study presented by Binnetoglu et al. (9) showed that 55 patients (83.33%) were term, 11 patients (16.67%) were preterm, and 22 patients (33.33%) were diagnosed in the prenatal period. In the present study, NA was more common in term infants, with an incidence of 81.01% (145/179). Our results were compatible with the literature (6, 9).

Electrolyte disturbances can affect the self-discipline, excitability, and conductivity of cardiac muscle cells, leading to NA (23). Kundak et al. (11) performed a retrospective observational study, and the result showed that 16 patients were complicated by electrolyte disorder, including nine cases of hyperkalemia (56.25%). In the study reported by Shortland et al. (24), hyperkalemia was associated with a high incidence of cardiac arrhythmia (60%). In the present study, the most common electrolyte disorder was hyperkalemia, which was mainly presented in patients with non-benign NA. Otherwise, in the present study, a 27 weeks' GA case presented SVT, septic shock, and hyperkalemia during hospitalization. Ultimately, the SVT stopped after antishock and correction of hyperkalemia, without recurrence of NA.

Benign NA usually presented rare clinical symptoms or recurrence or death. Nagashima et al. (25) reported that an incidence of PAC was 50.79% (32/63) in normal newborn infants, and it disappeared in the first few days without recurrence. In the present study, the incidence of PAC was 0.08% (38/47199). Otherwise, we also observed that 96.69% (117/121) of benign NAs did not require special treatment. Four cases of benign NA received antiarrhythmic therapy, including three premature contraction and one junctional arrhythmia. The patient with junctional arrhythmia had a recurrence once after discharge at 1 year old, and metoprolol was used for 6 months and then stopped.

Nonbenign NA needed active treatment because it could easily lead to hemodynamic instability and even life-threatening (26). The study by Binnetoglu et al. (9) demonstrated that the most common arrhythmias were supraventricular ectopic beats and SVT at 39.39 and 22.72%, respectively. SVT recurred in five patients after the neonatal period. Gilljam et al. (8) retrospectively reviewed 109 patients with SVT, and the result showed that the rate of freedom from arrhythmia, antiarrhythmic medication, or late recurrence of arrhythmia was 52% at 1 year. Our results were compatible with the literature (8, 9). In the present study, SVT was the most frequently observed type of non-benign NA (68.97%). Among 40 patients with SVT, 33 received antiarrhythmic therapy, and the recurrence rate was 50% at 1 year. Adenosine was the first choice for treating SVT, and the mechanism of action was related to AV node block (27). To prevent SVT recurrence, antiarrhythmic prophylaxis was recommended during the first year of life (2, 3).

AFL presents excellent long-term prognoses if actively treated. The study reported by Casey et al. (20) in 25 neonates with AFL showed that 84% of patients (21/25) received antiarrhythmic therapy, including digoxin and electrical cardioversion, without recurrence during long-term follow-up (median, 23 months). In the present study, 70% of patients (7/10) with AFL received antiarrhythmic therapy, nine survived during follow-up, and one died because of respiratory failure.

VT is rare in newborns, and death is also rare after active treatment of the primary disease. The study by Davis et al. (21) in 40 infants and young children with VT indicated that the most common causes were cardiomyopathy or myocarditis. Singh et al. (28) reported an extremely low birth weight neonate developing VT following hyperkalemia, and no recurrence was shown after treatment with lidocaine. In the present study, VT was presented in three patients induced by cardiomyopathy, severe electrolyte disturbance, and CHD. Of them, two patients received antiarrhythmic therapy, and one patient received electrical defibrillation.

Complete AV block induced by severe bradycardia could lead to low cardiac output and heart failure (9). Complete AV block in a normal structural heart might occur in infants born to mothers with connective tissue disorders such as systemic lupus erythematosus (2). A retrospective, multicenter study of 175 patients (22) indicated that AV block was associated with maternal anti-Sjögren syndrome antigen antibodies. In the present study, four infants presented second-degree AV block. But all of them did not receive treatment. Otherwise, one patient with a normal-structure heart was born to a mother with antinuclear/anti-double-stranded deoxyribonucleic acid antibodies.

The major limitations of the present study were as follows: (1) relatively small sample size in a single-center study, and (2) retrospective study might underestimate the incidence of NA, and mild and/or transient NA may not be recorded. These limitations might induce potential bias, including restricted application scope. These problems could be overcome in additional studies. Given the potential limitations, more trials are needed in the future.

## Conclusion

In summary, compared with neonates with benign arrhythmia, those neonates with non-benign arrhythmia showed higher rates of death and recurrence. In future studies, because of the high recurrence rate, more attention is needed in neonates with non-benign NA after discharge.

## Data Availability Statement

The raw data supporting the conclusions of this article will be made available by the authors, without undue reservation.

## Ethics Statement

The studies involving human participants were reviewed and approved by the Ethics Committee of Children's Hospital of Chongqing Medical University. Written informed consent from the participants' legal guardian/next of kin was not required to participate in this study in accordance with the national legislation and the institutional requirements.

## Author Contributions

LR collected the data and drafted and reviewed the manuscript. JL drafted and reviewed the manuscript. LB reviewed and revised the manuscript. LC conceptualized the study and reviewed and revised the manuscript. All authors approved the final manuscript as submitted and agree to be accountable for all aspects of the work.

## Funding

This work was supported by Natural Science Foundation of Chongqing (cstc2020jcyj-msxmX0197).

## Conflict of Interest

The authors declare that the research was conducted in the absence of any commercial or financial relationships that could be construed as a potential conflict of interest.

## Publisher's Note

All claims expressed in this article are solely those of the authors and do not necessarily represent those of their affiliated organizations, or those of the publisher, the editors and the reviewers. Any product that may be evaluated in this article, or claim that may be made by its manufacturer, is not guaranteed or endorsed by the publisher.

## References

[1] Tanel RE Rhodes LA. Fetal and neonatal arrhythmias. Clin Perinatol. (2001) 28:187–207. 10.1016/S0095-5108(05)70074-9 11265506

[2] Ban JE. Neonatal arrhythmias: diagnosis, treatment, and clinical outcome. Korean J Pediatr. (2017) 60:344. 10.3345/kjp.2017.60.11.344 29234357 PMC5725339

[3] Jaeggi EO Hman A. Fetal and neonatal arrhythmias. Clin Perinatol. (2016) 43:99–112. 10.1016/j.clp.2015.11.007 26876124

[4] Dubin AM. Arrhythmias in the newborn. Neoreviews. (2000) 1:e146–51. 10.1542/neo.1-8-e146

[5] Moura C Vieira A Guimares H Areias JC. Perinatal arrhythmias–diagnosis and treatment. Rev Port Cardiol. (2002) 21:45–55. 11941900

[6] Badrawi N Hegazy RA Tokovic E Lotfy W Mahmoud F Aly H. Arrhythmia in the neonatal intensive care unit. Pediatr Cardiol. (2009) 30:325–30. 10.1007/s00246-008-9355-4 19184182

[7] Picchio FM Prandstraller D Bronzetti G Cervi E. Follow-up of neonates with foetal and neonatal arrhythmias. J Matern Fetal Neonatal Med. (2012) 25:53. 10.3109/14767058.2012.714980 22958016

[8] Gilljam T Jaeggi E Gow RM. Neonatal supraventricular tachycardia: outcomes over a 27-year period at a single institution. Acta Paediatr. (2008) 97:1035–9. 10.1111/j.1651-2227.2008.00823.x 18489621

[9] Binnetoglu FK Babaoglu K Turker G Altun G. Diagnosis, treatment and follow up of neonatal arrhythmias: cardiovascular topic. Cardiovasc J Afr. (2014) 25:58–62. 10.5830/CVJA-2014-002 24844549 PMC4026762

[10] Isik DU Celik IH Kavurt S Aydemir O Kibar AS Bas AY. A case series of neonatal arrhythmias. J Matern Fetal Neonatal Med. (2016) 29:1344–7. 10.3109/14767058.2015.1048679 26037725

[11] Kundak AA Dilli D Karagöl B Karadag N Zenciroglu A Okumuş N. Non benign neonatal arrhythmias observed in a tertiary neonatal intensive care unit. Indian J Pediatr. (2013) 80:555–9. 10.1007/s12098-012-0852-3 23054850

[12] Strasburger JF Cheulkar B Wichman HJ. Perinatal arrhythmias: diagnosis and management. Clin Perinatol. (2007) 34:627–52. 10.1016/j.clp.2007.10.002 18063110 PMC3310372

[13] Wren C. Cardiac arrhythmias in the fetus and newborn. Semin Fetal Neonatal Med. (2006) 11:182–90. 10.1016/j.siny.2005.12.001 16530495

[14] Phelan JP Ahn MO Smith CV Rutherford SE Anderson E. Amniotic fluid index measurements during pregnancy. J Reprod Med. (1987) 32:601–4. 10.1007/BF01533766 3309290

[15] Subspecialty Subspecialty Group of Neonatology the the Society of Pediatric Chinese Medical Association Professional Professional Committee of Infectious Diseases Neonatology Society Chinese Medical Doctor Association. Expert consensus on the diagnosis and management of neonatal sepsis (Version 2019). Chin J Pediatr. (2019) 57:252–7. 10.3760/cma.j.issn.0578-1310.2019.04.005 30934196

[16] Freedom RM Benson LN Smallhorn JF. Neonatal Heart Disease. London: Springer (1992). 10.1007/978-1-4471-1814-5

[17] Cecconi M Debacker D Antonelli M Beale R Bakker J Hofer C. Consensus on circulatory shock and hemodynamic monitoring. Task force of the European Society of Intensive Care Medicine. Intens Care Med. (2014) 40:1795–815. 10.1007/s00134-014-3525-z 25392034 PMC4239778

[18] Mou JF Pan XR Chen BL Pan XN. Comparison of clinical characteristics of benign versus non-benign neonatal arrhythmia. Guangxi Med J. (2020) 9:1072–5.

[19] Wang YY Kang WQ Zhang YD Liu DT Fang PP. Clinical analysis of neonatal arrhythmia in 89 cases. J Chin Pract Diagn Ther. (2018) 9:896–8. 10.13507/j.issn.1674-3474.2018.09.019

[20] Casey FA McCrindle BW Hamilton RM Gow RM. Neonatal atrial flutter: significant early morbidity and excellent long-term prognosis. Am Heart J. (1997) 133:302–6. 10.1016/S0002-8703(97)70224-2 9060798

[21] Davis AM Gow RM McCrindle BW Hamilton RM. Clinical spectrum, therapeutic management, and follow-up of ventricular tachycardia in infants and young children. Am Heart J. (1996) 131:186–91. 10.1016/S0002-8703(96)90068-X 8554007

[22] Eliasson H Sonesson SE Sharland G Granath F Hanséus K. Isolated atrioventricular block in the fetus: a retrospective, multinational, multicenter study of 175 patients. Circulation. (2011) 124:1919–26. 10.1161/CIRCULATIONAHA.111.041970 21986286

[23] El-Sherif N Turitto G. Electrolyte disorders and arrhythmogenesis. Cardiol J. (2011) 18:233–45. 21660912

[24] Shortland D Trounce JQ Levene MI. Hyperkalaemia, cardiac arrhythmias, and cerebral lesions in high risk neonates. Arch Dis Child. (1987) 62:1139–43. 10.1136/adc.62.11.1139 3688918 PMC1778541

[25] Nagashima M Matsushima M Ogawa A Ohsuga A Kaneko T Yazaki T. Cardiac arrhythmias in healthy children revealed by 24-hour ambulatory ECG monitoring. Pediatr Cardiol. (1987) 8:103–8. 10.1007/BF02079464 2442731

[26] Yildirim SV Tokel K Saygili B Varan B. The incidence and risk factors of arrhythmias in the early period after cardiac surgery in pediatric patients. Turk J Pediatr. (2008) 50:549. 10.1053/j.sempedsurg.2008.07.008 19227418

[27] Camm AJ Garratt CJ. Adenosine and supraventricular tachycardia. New Engl J Med. (1991) 325:1621–9. 10.1056/NEJM199112053252306 1944450

[28] Singh D Dutta S Narang A. Hyperkalemia and ventricular tachycardia in ELBW infant. Indian Pediatr. (2003) 40:64–6. 12554924

